# Are spatial frequency cues used for whisker-based active discrimination?

**DOI:** 10.3389/fnbeh.2014.00379

**Published:** 2014-11-03

**Authors:** Petya Georgieva, Dominik Brugger, Cornelius Schwarz

**Affiliations:** ^1^Systems Neurophysiology, Werner Reichardt Center for Integrative Neuroscience, University TübingenTübingen, Germany; ^2^Department for Cognitive Neurology, Hertie-Institute for Clinical Brain Research, University TübingenTübingen, Germany

**Keywords:** intracortical electrical stimulation, behavioral modification, head-restraint rat, barrel cortex, whisking, intensity, instantaneous kinematic parameters

## Abstract

Rats are highly skilled in discriminating objects and textures by palpatory movements of their whiskers. If they used spatial frequency cues, they would be able to optimize performance in a stimulus dependent way—by moving their whisker faster or slower across the texture surface, thereby shifting the frequency content of the neuronal signal toward an optimal working range for perception. We tested this idea by measuring discrimination performance of head-fixed rats that were trained to actively sample from virtual grids. The virtual grid mimicked discrete and repetitive whisker deflections generated by real objects (e.g., grove patterns) with single electrical microstimulation pulses delivered directly to the barrel cortex, and provided the critical advantage that stimuli could be controlled at highest precision. Surprisingly, rats failed to use the spatial frequency cue for discrimination as a matter of course, and also failed to adapt whisking patterns in order to optimally exploit frequency differences. In striking contrast they could be easily trained to discriminate stimuli differing in electrical pulse amplitudes, a stimulus property that is not malleable by whisking. Intermingling these “easy-to-discriminate” discriminanda with others that solely offered frequency/positional cues, rats could be guided to base discrimination on frequency and/or position, albeit on a lower level of performance. Following this training, abolishment of electrical amplitude cues and reducing positional cues led to initial good performance which, however, was unstable and ran down to very low levels over the course of hundreds of trials. These results clearly demonstrate that frequency cues, while definitely perceived by rats, are of minor importance and they are not able to support consistent modulation of whisking patterns to optimize performance.

## Introduction

Sensory perception is an active process with a reciprocal interplay between the read out of incoming sensory signals and the active sensing strategy of the subject. The rodent whisker-related sensorimotor system is outstanding as a model system because these animals use their mobile whiskers to “actively scan” their environment (Carvell and Simons, 1990). Active scanning goes beyond focusing and directing sensor organs to sources of sensory signals as in hearing and seeing. It means that rodents deploy energy to objects (via whisker movements) and gain information by sensing the object’s reflections (in form of fine object-dependent whisker vibrations) (Hentschke et al., 2006; Ferezou et al., 2007; Wolfe et al., 2008). This is akin to active scanning using echo-location or electro-sensation of cetaceans and fish, and is performed by humans in very similar ways using their finger tips (Gamzu and Ahissar, 2001). It is comprehensible that active scanning will emphasize the above mentioned sensorimotor interplay as any change in movement strategy fundamentally changes the character of incoming sensory sensation and vice versa.

In the laboratory setting, rats are able to perform fine object and texture discrimination tasks using whisker touch of surfaces (Carvell and Simons, 1990; Harvey et al., 2001), and there is initial evidence that rats adapt their whisking strategy in a problem oriented manner (Carvell and Simons, 1995). In order to gain a deeper understanding of active scanning it is decisive to firstly understand which aspect of the vibrotactile signal (the fine object related vibrations) is encoded in the tactile system. In a second step it needs to be clarified how scanning movements change these critical parameters. By locating the encoded signals in the neuronal tactile system one can hope to find the mechanism by which they are transformed when adapting whisking patterns. The candidate aspects of vibrotactile signals which the animal could use for active perception fall roughly into three classes—frequency (any result from analysis of the frequency domain representation of the tactile signal), intensity (anything that bases tactile decisions on temporal integrals of kinematic time series) or kinematic events (anything that uses distributions of highly resolved kinematic time series as basis for perception—or, in the extreme, relies on the single occurrence of short informative trajectory snippets). For instance, in case of the commonly used pulsatile tactile stimuli, frequency is commonly approximated by pulse frequency (Salinas et al., 2000), intensity by mean speed, power or any other temporal integration of the signal (Arabzadeh et al., 2003), and instantaneous kinematics by the distribution of amplitudes, velocities, (or any other kinematic parameter) contained in the tactile stimulus (Gerdjikov et al., 2010; Waiblinger et al., 2013). The importance of frequency and intensity, the first two variables, has been suggested by classic studies in the primate hand/finger system using sinusoidal skin deflections (LaMotte and Mountcastle, 1975). Sinusoids, however, do not allow to disentangle the three groups of variables, as instantaneous kinematic parameters (e.g., the maximum velocity of a sine wave or the waveform of one period) are changed concomitantly with frequency. Pulsatile stimuli resolve this problem as pulse waveforms (i.e., kinematic events) and inter-pulse intervals (i.e., frequency) can be altered independently. Rats trained on a task to discriminate such stimuli, while holding their whiskers still, use predominantly the instantaneous kinematic cues and largely ignore frequency and intensity cues (Waiblinger et al., 2013).

The question which of the three groups of vibrotactile parameters is encoded touches on the more general question which role temporal integration plays for perception. The analysis of frequency and intensity require extensive temporal integration while the detection of instantaneous kinematic events (i.e., trajectory snippets), in the extreme, may require no integration time at all. There can be no doubt that increasing integration time often helps to improve perception. However, in most behavioral circumstances time and accuracy are traded against each other (Roitman and Shadlen, 2002). Analysis of instantaneous kinematic parameters is advantageous, because it saves time. On the other hand, whenever cognitive processing is required to guide behavior, a speedy decision may often be less important than to identify the correct behavioral strategy. In this case, temporal signal integration may be employed to compress tactile information into a small format adequately required to handle and store the signals in memory for decision making (Gerdjikov et al., 2010; Feldmeyer et al., 2013). At first glance, active sampling of tactile signals seems to fall largely in the second category. Tactile signals are sampled using multiple whisker strokes occurring at about 10 Hz. These eventually non-continuous periods of tactile signals firstly have to be connected to generate a unique percept, and secondly, must be bound into meaningful sequences of other body movements. Thus, active discrimination doubtlessly requires executive control which stores tactile signals in memory and generates (bases on these memory contents and other contextual parameters) the sequencing of behavioral elements in a goal oriented way. Therefore, we expected that active discrimination might employ temporal integration of vibrotactile signals.

To find out which cues are used in the case of active sampling two problems arise. The first is that stimulus control is difficult to achieve, because freely running animals have multiple degrees of freedom in choosing the ways to probe a texture. Secondly, the frequency cue is difficult to isolate from other cues as the waveform of the vibrotactile signal is entirely controlled by the animals and not the experimenter. To remedy the first problem we trained head-fixed rats to move a whisker (Hentschke et al., 2006; Gerdjikov et al., 2013) across a so called “virtual grid”, composed of a series of single-pulse intracortical electrical stimulation (ICMS), whenever the rat’s whisker crossed certain positions (the virtual grid points). This arrangement seems artificial, but, firstly, we gain excellent stimulus control and great specificity for the grid parameters spatial frequency as well as position, decisive for the questions we ask here. Secondly, single ICMS pulses evoke neuronal signals in barrel cortex (Butovas and Schwarz, 2003, 2007; Butovas et al., 2006) that are not unlike responses seen after a brisk whisker deflection (Simons and Carvell, 1989; Moore et al., 1999; Hentschke et al., 2006; Stüttgen and Schwarz, 2008): single action potentials or short high frequency bursts followed by an inhibitory period.

Our first approach with this method was to ask whether spatial frequency cues are used in active discrimination. We further were interested to find out whether rats would adapt their whisking strategy to change the conversion of spatial stimulus frequency to temporal neuronal frequency, in order to optimize discrimination. This expectation was not met—rats struggled to use frequency (and position) cues and their whisking pattern did not change systematically. Interestingly, it was electrical pulse amplitude that allowed the animals to discriminate virtual grids best. Because electrical pulse amplitude changes are similar in effect as modulation of velocity of short whisker deflections, we interpret these results as supporting the idea, gained from results on passive discrimination (Waiblinger et al., 2013), that instantaneous kinematic cues are also used predominantly when acquiring the vibrotactile signal using active scanning.

## Materials and methods

Thirteen male Sprague Dawley rats were used in this study. Ten were used in preliminary experiments in which the superiority of amplitude cues over frequency cues was found. Based on this insight we report performance of three further rats on a series of detailed psychophysical tests which aimed to emphasize a contribution of frequency or position, if present. All procedures were conducted according to German law and the rules set by the Society for Neuroscience. The procedures to handle the animals, head-cap and electrode implantation, water control, habituation to head fixation, tracking whisker movement of a single whisker, and monitoring licking were done exactly as published previously (Schwarz et al., 2010). In the following we will go into details only where we digressed from those methods.

Rats were habituated to the experimenter for 2 weeks and then underwent head-cap surgery after a 2 day treatment with antibiotics (Baytril^®^ 2.5%, oral solution, Bayer, 500 ml). For the surgery the rats were anesthetized with an initial dose of ketamine (100 mg/kg) and xylacine (15 mg/kg) delivered i.p. followed by ventilation with 2% isofluran (in medical oxygen). Before inserting the electrodes the position of the C1 barrel column in the primary somatosensory cortex was estimated using electrophysiological mapping while manually deflecting single whiskers. A 2 × 4 movable microelectrode array (impedance > 1 MΩ) was implanted centered on the C1 barrel column. The electrode tips were lowered to 250–300 µm cortical depth via a micromanipulator (later in the awake animal, just before microstimulation commenced, they were moved to a depth of 1000 µm). A fixation screw was embedded head down in the caudal part of the implant (over the bone overlying the cerebellum). At the end of the surgery pain medication was commenced (caprofen 5 mg/kg, two times daily, for 3 days). Further, local antibiotic and 5 ml Glucose (5%) was administered. The animals were allowed to regenerate for minimally 10 days before habituation to head fixation started under water control lasting 10–14 days.

**Figure 1:**
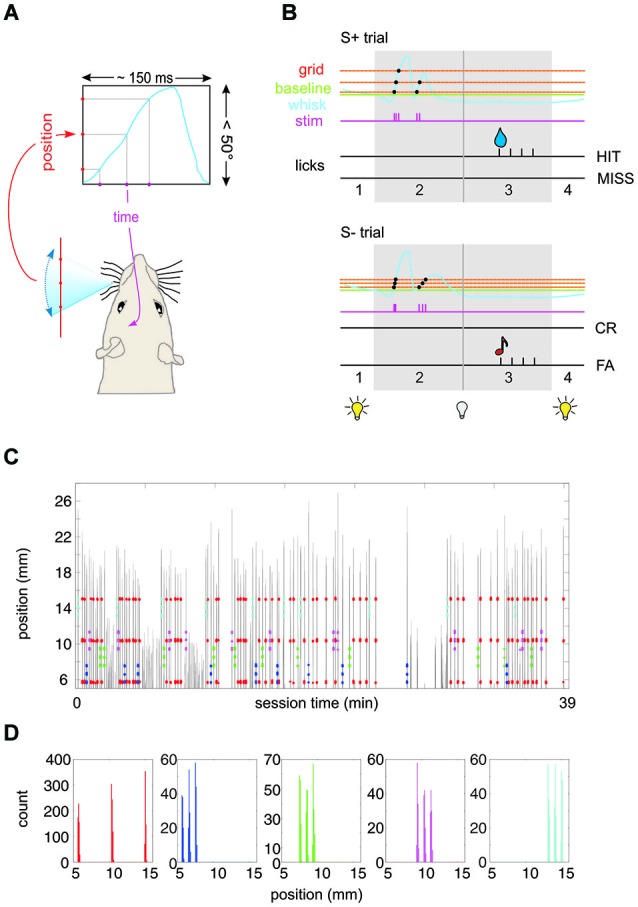
**Experimental paradigm**. **(A)** Virtual grid. A real-time algorithm assessed the position of the whisker and generated an intracortical microstimulation (ICMS) pulse to the barrel cortex of the head-fixed rat whenever the whisker passed a pre-defined position. The blue curve depicts schematically one period of whisker movement. The virtual environment converts the spatial frequency of the grid (red) into the temporal frequency of the ICMS pulse train (purple). **(B)** Schematic of the behavioral paradigm. The house light was on as long as the whisker position was in front (protracted to) the base line (broken green line) (phase 1). Once the whisker retracted behind this line the house light went off to signal trial start. The rat had 1 s to sample the virtual grid (phase 2). Protraction movements (blue) across the grid (red) would activate single ICMS pulses (purple). After this period phase 3 allowed the rat to retrieve water reward (droplet) by licking from the spout in case of S+ presentation (top). Depending on whether the rat licked the trial was classified as HIT or MISS trial. In case of S− presentation (bottom), a lick was counted as false alarm (FA) and followed by a tone. No lick was classified as correct rejection (CR). After the indicator interval (phase 3) the house light went on to indicate the end of the trial. **(C)** Spatial precision of virtual grid. Example session of rat 1 working on Test 3 (cf. Figure 3). Whisker movement across the laser optical device (LOD) is shown (gray; the distance of the LOD is 2 cm from the face, i.e., 1 mm ≙ 2.86°). The position at which an electrical stimulation pulse was delivered to the barrel cortex is marked by colored dots (red: S+; blue/green/magenta/cyan: S1−/S2−/S3−/S4). **(D)** Same session as shown in **(C)**. Here the distribution of positions at which a stimulation pulse occurred is plotted for the five different stimuli (colors correspond to panel **C**). The spatial spread of a virtual grid point measured in this way (across all stimulations in all rats) displays a standard deviation of 0.2°.

The well habituated rats were trained on a Go/No Go discrimination task that required them to move their whisker to sample virtual grids (i.e., a series of ICMS pulses via one of the implanted microelectrodes, generated by issuing a single pulse every time the tracked whisker crossed a virtual grid point; Figure 1A), decide whether the stimulus predicted reward (Go) or not (No Go), and indicating this decision by either emitting a lick at the water spout (Go) or refraining to lick (No Go; Figure 1B). The training steps to acquire the capability to perform this task were as follows: in a first step all animals were conditioned to passively detect electrical stimulation (Butovas and Schwarz, 2007). A series of biphasic ICMS pulses (5–15 biphasic electrical pulses, 30–50 µA at 60 Hz; single pulse duration 500 µs) were generated using a programmable stimulator (STG4001; Multi Channel Systems MCS GmbH) and coincided with the delivery of a water drop at a spout positioned in front of the animal’s snout. Next, water delivery was made contingent on a correct lick after the ICMS pulse. After acquiring most of the rewards the number of ICMS pulses was decreased step-by-step until the animals detected well a single ICMS pulse. The lowest pulse amplitudes used in the present study to test discrimination ability were detected as single pulses by the animals at minimal correct response rates of 0.8. At this point a laser optical device (LOD, Metralight Inc., San Mateo, CA, USA) was used to track the C1 whisker (inserted in a light weight polyimide tube) at a distance of 2 cm from the face (Hentschke et al., 2006). The remaining whiskers were trimmed to 1 cm. The output of the LOD was monitored in real time and used to make the delivery of the ICMS pulse contingent to the crossing of a virtual grid point. In the beginning, the virtual grid point was located close to the resting position of the whisker, but in subsequent sessions, depending on the animals performance, it was placed farther and farther rostral to it. The rat was only rewarded for a successful movement eliciting an ICMS pulse followed by a lick, never for movement and licking alone. At this training level all rats generated large amplitude whisks (>30°). In the final step, the house light was used as an indicator that a trial could be initiated by whisking across the starting point (typically the whisker’s resting position). “House light off” indicated that the whisker was in the correct position behind the start point and that trial initiation was enabled. “House light on” signaled that the whisker was located rostral to the starting line and trial initiation was disabled. At this stage the single pulse stimulation was replaced by a virtual grid of three equally spaced virtual points. A stimulation pulse was delivered at each crossing of a virtual point in the protraction direction. In this way, a spatial virtual object—the 3-point grid—was translated into the temporal frequency of stimulation delivered to the barrel cortex (Figure 1A). After a 1 s sampling period, in which the animal was free to probe the virtual grid using movements of its choice (but was required to generate at least one full sweep across all three virtual grid points), a “window of opportunity” interval came on, signaled by switching on the house light. If the sampled stimulus predicted reward (S+), a lick in the window of opportunity yielded the delivery of a drop of water as reward. If the stimulus did not predict reward (S−), a lick in this period triggered a tone to signal the error followed by a light punishment in the form of a 2 s time out (Figure 1B). Subsequently, the next trial could be initiated any time given an interval of 200 ms had passed after the last lick. The rats were free to lick while sampling the virtual grid with no consequence. S+ and S− were presented in pseudo-random fashion. In all tests the overall ratio of S+/S− trails was 1:1. In some tests the group of S+ or S− trials were divided between many stimuli.

**Figure 2:**
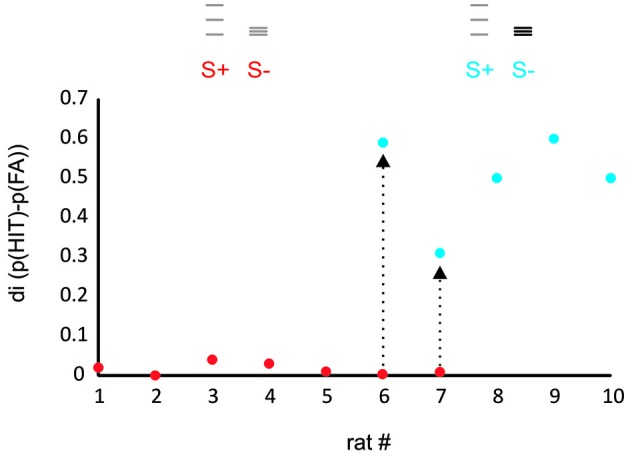
**Rats learn to discriminate ICMS amplitude but not spatial frequency**. The plot shows the discrimination index (di, the difference between relative frequency of HIT and FA) for a task presenting virtual grids that differed in spatial frequency and range of grid bar positions (red dots, task 1) and a task that in addition offered differences in ICMS amplitude (blue dots, task 2). Rats 1–5 were trained exclusively with the first stimulus set and never learned the task. Rats 6, 7, after showing similar results on the first task were switched to task 2 and learned the task within several sessions (arrows). Rats 8–10 were trained only to task 2 and readily learned it. The icons depict the position of the virtual grid bars, gray: small ICMS amplitude, black: large ICMS amplitude.

**Figure 3:**
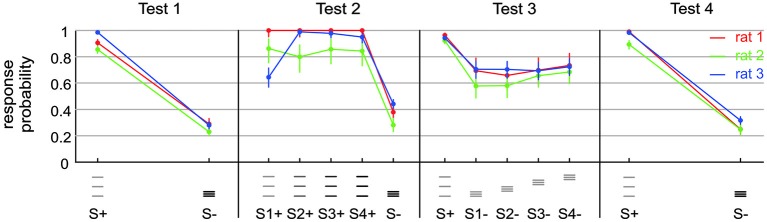
**Exposing a small capability of rats to discriminate spatial frequency**. Test 1 presented stimuli containing frequency and amplitude cues (S+: af; S−: AF). Test 2 presented the same stimuli (S1+: af; S−: AF) but also amplitude matched ones (S4+: 4af = Af), as well as intermediate stimuli (S2+: 2af; S3+: 3af). Note that FA rate increased a bit, but Af/AF discrimination is comparable to af/AF discrimination in this context. Test 3 removed the amplitude cues from the set of discriminanda (S+: af; S1− to S4−: aF) and blurred the positional cue by varying the range of virtual grid bar positions. A small remaining overall capability to discriminate the set of discriminanda is visible. The result on Test 4 is identical to Test 1. This control shows that the general motivation of the rats to perform on the tasks was still high after performing on Test 2 and 3.

**Figure 4:**
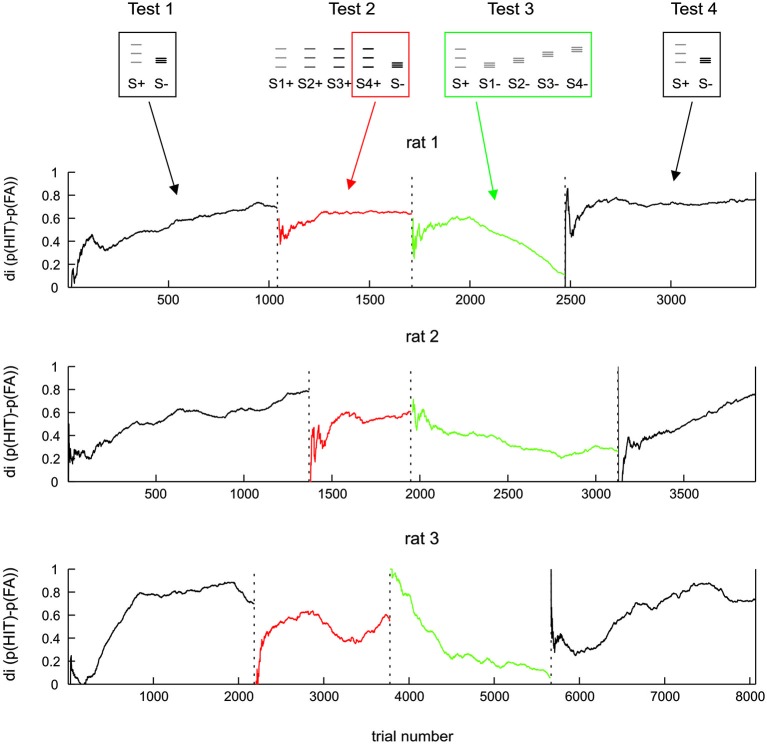
**Same data as Figure 3 but shown along trials after boxcar filtering (each Test data was filtered separately; width 500 trials)**. For test 2 the discrimination of the amplitude matched pair is shown (boxed icons on top).

**Figure 5:**
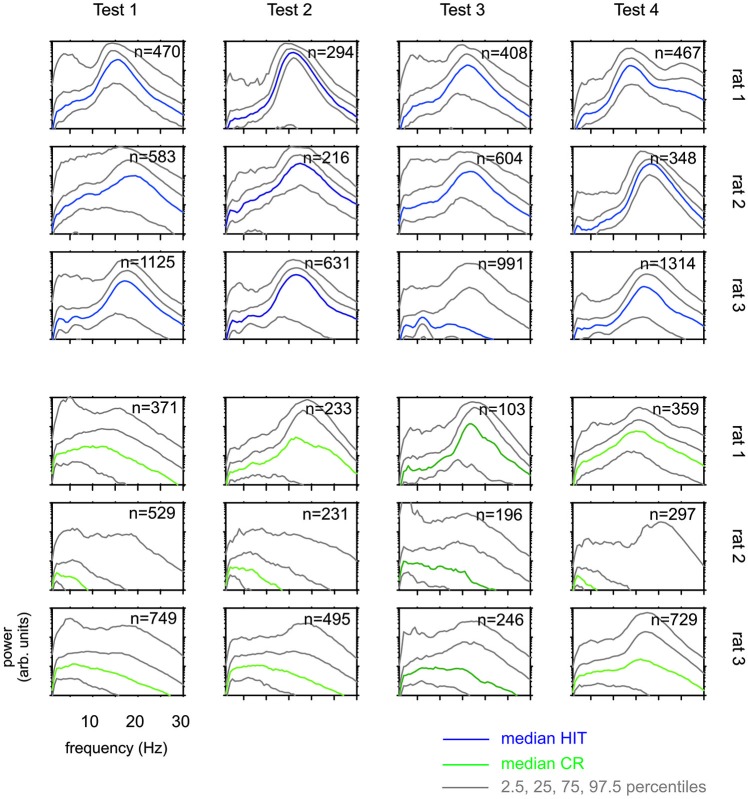
**Whisking patterns for each rat when performing on Test 1 to Test 4.** Distributions of power spectra obtained during the 1 s sampling period (phase 2 in Figure 1B) HIT trials (blue, top) and correct rejection (CR) trials (green, bottom). Shown are the median (colored) and the percentiles as indicated in the legend. There are systematic differences across rats. Across Tests, however, each animal generated similar whisking patterns.

**Figure 6:**
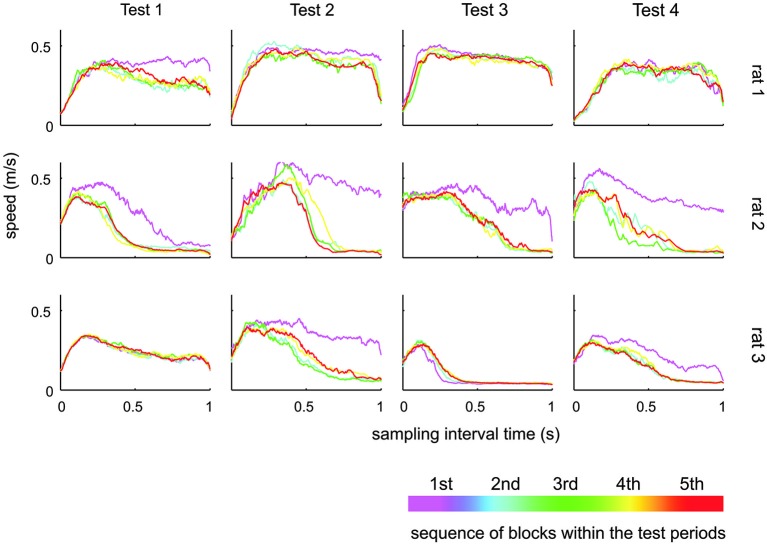
**Whisker speed during sampling interval**. Shown is the average whisker speed across time split into five subsequent blocks during the respective test. During the first block of a new test, the animals tended to generate higher whisker speeds. There was, however, no systematic change of whisking speeds across the tests.

For the acquisition of the final behavioral data reported in Figures 3–6, the three rats were tested in four subsequent psychophysical tests (Test 1 to Test 4) presenting sets of S+ and S− stimuli that varied in (1) spatial frequency; and (2) ICMS pulse amplitude. A third parameter was position which was necessarily different between S+ and S− because we had to refrain from presenting more than three virtual points per grid to avoid kindling of the brains. Spatial frequency could be either high (0.38/°, tagged “F”) or low (0.08/°, tagged “f”). In Test 1, 2 and 4, the position of the low frequency grid was [0.3 13.4 25.2]°, and that of the high frequency grid was [0.3 2.9 5.6]°. In Test 3 the high frequency grids were presented at three additional positions [5.6 8.2 10.9], [10.9 13.5 16.2], and [19.9 22.5 25.2]° (all positions with respect to the starting line). To demonstrate the precision of imprinting the virtual grid Figures 1C,D present an example whisker trace of one session acquired during presentation of Test 3. The gray lines are whisker position traces during sample periods throughout session time. The colored dots indicate the whisker positions at which an electrical stimulus was applied (S+: red; S1−: blue; S2−: green; S3−: magenta; S4−: cyan). Panel D shows the distribution of whisker positions at which stimulation occurred during this example session (rat 1). The spatial distribution of virtual grid points is characterized by three well separated narrow peaks. We analyzed the spatial variability of all 577 peaks obtained in this way from the three rats in Test 3 and found a standard deviation of 0.2°, i.e., (assuming normality) 96% of virtual grid points were located within ± 0.4° of the locations given above. The ICMS amplitudes were adjusted individually for each rat. Rat 1 and 3 received 30 µA and 150 µA, while Rat 2 received 50 µA and 200 µA. The high and low current amplitudes were tagged “A”, and “a”, respectively.

**Test 1** was to discriminate between just two grids, differing in spatial frequency and amplitude. S+ was af and S− was AF. We had to test the animals first on this task, because, as will be detailed in the results, stimuli offering exclusively frequency cues could not be discriminated well enough by the animals to learn the tasks contingencies.

**Test 2** was designed to find out in how far the animals can use stimuli that differed in frequency (and position) only. One of the S+ and the sole S− were af and AF, respectively, exactly as in test 1. In addition, however, we presented 2af, 3af, and 4af, with 4a being equal to A, i.e., identical in pulse amplitude to the S+. In this test our main focus was whether the animals could discriminate between Af(= 4af) and AF (Rat 1 and 3: [a, 2a, 3a, 4a] = [30, 70, 110, 150] µA; Rat 2: [a, 2a, 3a, 4a] = [30, 70, 110, 150] µA).

**Test 3** was designed to find out if remaining discrimination performance found with test 2 was due to the different positions. The S+ was af and four different S− (all aF) were presented at different positions (see above).

**Test 4** was a control that used (as already done in Test 1) both, spatial frequency and current amplitude as cues. Rat 1 readily performed on a stimulus set that was like the one used in Test 3 but offered amplitude cues S+: af. S−: AF. Rats 2 and 3 were put on the simpler Test 1 again.

The experiment was controlled automatically, without any experimenter input, by custom written software in LabView RealTime using a National Instruments I/O card (National Instruments, Austin,TX, USA). The real time software ran at a duty cycle in which a position measurement was taken and a trigger signal for stimulation (if required) was issued. The timing of the duty cycle was monitored and an error was generated if it exceeded 0.4 ms. The present data were sampled exclusively with runs that successfully kept this cycle time. All behavioral data, whisker trace, licking events, and all experimentally controlled timestamps (trial periods, pulse delivery) were written to a binary file and analyzed using custom written Matlab scripts (Mathworks, Natick, MA, USA).

## Results

We trained head fixed rats to sample a three-point virtual grid by whisking in free air and to indicate their decision whether they perceived a grid that predicted (Go), or did not predict (No Go) reward by licking or refraining to lick at a water spout. Virtual grids were presented by virtue of real-time tracking of whisker movement and delivering a single ICMS pulse to the barrel column C1 immediately after the whisker passed a virtual grid point (Figure 1A). The animals self-initiated a trial by bringing the whisker behind a start point and protracting it towards the location of the virtual grid. A 1 s interval time opened that allowed to sample the presented virtual grid using whisker movements of the rats’ choice. A trial was counted as valid if at least one full crossing of the whole virtual grid had been done in this 1 s interval. The animal then had another second to indicate its response (Figure 1B).

The performance of the animals was estimated for each training session by calculating a discrimination index (di) which was defined as the difference of hit and false alarm (FA) rates observed in a session. In preliminary experiments, we attempted to train seven rats to discriminate a stimulus pair that solely differed in the position and spatial frequency of grid lines but was identical in ICMS pulse amplitude (red dots in Figure 2). None of these animals learned the task. In the last two of them, we introduced differences in pulse amplitudes which enabled the animals to learn the task in the course of a few hundred trials. Three further rats were trained using amplitude differences from the start, all of which learned the task (blue dots in Figure 2).

The result from these preliminary rats clearly indicated that the animals did not make use of the frequency cues as a matter of course. Most likely therefore, spatial frequency converted into temporal frequency of barrel cortex activation is not a dominant perceptual cue. The straight negative result, however, did not let us gauge whether the difficulty was absolute, i.e., whether the animals were not able to use frequency cues at all, or alternatively, that they perceived it to some extent but in the context of the complex behavioral task could not make use of it. In a set of three animals we set out to perform a detailed behavioral analysis aimed at revealing the discrimination capability of rats based on frequency cues—if there was any. We reasoned that after learning the secondary task contingencies (putting the whisker behind the start line, respect sampling time and wait for the response window, etc.), the rats should be free to employ their perceptional resources to use frequency/position as the discriminant cue and thus may master also what we call the primary contingency—that of stimulus frequency/position and reward. We, therefore, trained them initially to discriminate stimuli that differed in frequency/position as well as electrical pulse amplitude, a task that had been successfully tackled by the rats in the preliminary experiments (Figure 3, Test 1 identical to the second experiment depicted using blue symbols in Figure 2). Expectedly, all three rats learned the task. The HIT rates, i.e., probability of response to the S+ stimulus (af = small amplitude, small frequency, see methods and icons in Figure 3), was far higher than 0.8, and the FA rate (i.e., the probability to respond falsely to S− which was AF = high amplitude, high frequency) stayed below 0.3. After thus mastering the secondary contingencies, we set out to confront the animals with stimuli that were matched in pulse amplitude (Figure 3, Test 2). Preliminary tests, however, showed a strong deterioration of performance using exclusively stimulus pairs with matched amplitudes. We therefore, presented four variants of the S+ stimulus (S1+ to S4+) varying systematically the electrical pulse amplitude. S1+ was identical to S+ of Test 1 to provide the animals with a motivating “easy” discrimination, while S4+ displayed identical electrical pulse amplitude as S−. The remaining two stimuli (S2+ and S3+) showed pulse amplitudes interspersed between these extremes. Compared to Test 1, discrimination performance in Test 2 was slightly impaired as indicated by the somewhat elevated response rates to S− (which was AF, to around 0.4). Importantly, however, the animals could keep a fairly high HIT rate to all stimuli (mostly above 0.8), including the stimulus matched in amplitude, suggesting that we were able to demonstrate a certain capability of rats to use frequency or position as a perceptual cue. This was further shown by Test 3 in which we finally abolished any amplitude cue, and secondly, blurred the positional cue by presenting the S− at varying positions within the confines of the lower frequency S+. All rats could keep a low overall level of performance with hit rates above 0.9, but FA rates approaching 0.7 (Figure 3, Test 3), indicating that the rats developed high impulsivity during this task probably associated with the perceptual difficulty. The control block (Figure 3, Test 4) showed that this deterioration of performance was not related to the rats’ motivation or any other general issue that would cause disengagement from the task. All rats were able to pick up the high level of performance shown in Test 1 once amplitudes cues were reintroduced (Figure 3, Test 4).

In order to investigate in more detail the ability of rats to use the frequency cue, we quantified discrimination performance over trials, calculating the di in a running average fashion from 500 trials surrounding each data point. This approach was made possible by the fact that Test 1 to 4 were presented in a blocked sequence, the switch from one test period to the next was abrupt with no interim arrangement to adapt animals to the next task (running averages were calculated separately within each test period, such that trials of one block did not affect performance presented for the adjacent block). The only difference between the procedures in different animals was that the number of trials performed in each test period was different to accommodate the build-up of task performance of each individual (Figure 4). The dynamics of di were found to be highly consistent across the three animals. All animals learned Test 1 within a few hundreds of trials (black lines). In Test 2, the di calculated from S4+ (the one that matched the electrical amplitude of S−, red lines in Figure 4) starts from a very low level, but then recovered to a relatively good level of discrimination, which however, was consistently lower than that seen toward the end of Test 1. After the switch to Test 3, which abolished all amplitude cues, the performance was either unaffected (rat 1 and 2) or even improved (rat 3). However, in the new context of the rather difficult Test 3 (characterized by a complete absence of amplitude cues and heavily blurred positional cues), none of the animals could uphold the relative good performance: after a few hundred trials di dropped gradually to a level of around 0.2 in all animals (green lines). Switching back to stimulus pairs offering amplitude cues (Test 4), the di of all animals recovered back to control levels (black lines).

The results presented so far suggest that rats have difficulties to base their discrimination solely on the frequency of actively sampled cortical ICMS, but guiding them using specific learning steps and task contexts, they can learn to use them to a certain extent. Next, we were interested if this ability is related to systematic changes in the movement patterns generated by the animals to sample the grids. Power spectra of whisker velocity traces during the sampling periods obtained in HIT and correct rejection (CR) trials during periods of Test 1 to 4 showed some variation with respect to the individual rat but were not found to systematically vary across Test periods in each rat (Figure 5). In an attempt to reveal whether whisking patterns relate to the dynamics of performance presented in Figure 4, we subdivided the test periods into five blocks holding equal numbers of trials. From each of these sub-blocks we calculated the progression of whisking activity within the sampling period in HIT trials as low pass filtered whisker speed (absolute whisker velocity convolved with a boxcar filter of 50 ms duration). The results (Figure 6) did show some adaptation of the whisking pattern in the first block of each test period—typically the rats started to work on a new test using high velocity whisking which extended well into the sampling interval. In later blocks whisking settled to lower speeds and shorter activity times. We observed different patterns for each test. For instance rat 3 showed extended whisking during the sample period in Test 1 which was then reduced in duration and amplitude in Test 2 and 3. However, these characteristics of adaption to the tests were not consistent across individuals. Rat 1, in a way, showed the opposite tendency. It extended whisking activity in time and increased whisking speed. Particularly, the poor showing on Test 3 observed in all rats (cf. Figure 4), was not accompanied by one common establishment of whisking pattern. The overall pattern during Test 3 was diversely characterized by higher whisker velocities in rat 1, more extended patterns in rat 2 and reduced velocity and extension in rat 3. Further, the slow progression toward poor performance from partly high rates at the beginning of Test 3 was not reflected in the progression of whisking patterns. On the contrary, the whisking patterns were surprisingly stable (if one excludes the adaptation described above—typically occurring in the first block of each test period). The inconsistency between task performance and whisking pattern was generally true for all tests and all rats.

## Discussion

Our results demonstrate that rats show a limited ability to discriminate the spatial frequency of actively acquired virtual stimuli. Consequently, they did not systematically adapt whisking patterns consistent with the optimization of frequency discrimination. In contrast, discrimination performance assumed very high levels when providing ICMS amplitude cues.

The present strategy to compare different potential coding schemes critically required highest stimulus control which was achieved by converting whisker touch with a virtual object into ICMS pulses. It needs to be clarified, therefore, whether the assumed artificiality of neuronal responses to ICMS precluded the usage of frequency for discrimination. The general applicability of ICMS to evoke behavioral responses has been shown repeatedly. On the neuronal level, ICMS was shown to readily activate local and distant cortical areas (Butovas and Schwarz, 2003; Tolias et al., 2005; Ferezou et al., 2007). Natural-like motor responses can be readily evoked with long trains of stimulation (Haiss and Schwarz, 2005; Graziano, 2006). However, brain stem pattern generators are likely involved in mediating ICMS evoked patterns of motor cortex activity to motoneurons in the brainstem/spinal cord (Chakrabarti and Schwarz, 2014). Perceptional effects have been shown repeatedly in visual cortex of humans and monkeys (Salzman et al., 1992; Schmidt et al., 1996; Bartlett et al., 2005) and somatosensory cortex of monkeys and rodents (Romo et al., 1998, 2000; Butovas and Schwarz, 2007). Our present findings corroborate these results as rats could readily discriminate the amplitude (Test 1) and for a short transient period also the frequency of ICMS (Test 3).

Are barrel cortex signals normally needed for perception? If yes, the present behavioral results, based on evoked barrel cortex activity, are able to inform us about characteristics of the neuronal basis of rat tactile perception. If not, the results would merely indicate which patterns of barrel cortex activity have access to perception. There is good evidence to support the notion that cortical tactile signals are normally required to accomplish the task and that therefore the experimental activation of barrel cortex is able to tap into basic requirements of tactile percepts. Operantly conditioned passive vibrotactile whisker-related detection (Sachidhanandam et al., 2013) and discrimination (Miyashita and Feldman, 2013) as well as actively perceived object and edge location (Hutson and Masterton, 1986; O’Connor et al., 2010) are abolished after cortical blockade. These cited studies have in common that cognitive processing plays a prominent role to accomplish the tasks—and therefore handling of tactile signals in memory networks is required. It is interesting to note that delay classically conditioned suppression tasks, which are more simply based on the frequency of paired and temporally overlapping stimulus presentations (Clark and Squire, 1998), allow detection and discrimination performance even after cortical lesions (Hutson and Masterton, 1986). However, in view of the above mentioned vital contribution of barrel cortex for operantly conditioned passive and active perceptual decision making, there is little reason to expect that performance on the operantly conditioned active discrimination task, used in the present study, would require barrel cortex less.

We hold that, for the purposes of mimicking tactile inputs to barrel cortex, ICMS activation may be far less artificial than appears at first glance. Whiskers typically touch objects very shortly (Mitchinson et al., 2007) and thus only transiently evoke activity on the tactile pathway. Due to biomechanical properties of the whisker leading to characteristic slip events, this is even true for prolonged contact with a textured surface (Arabzadeh et al., 2005; Ritt et al., 2008; Wolfe et al., 2008). Typically, these naturally occurring temporally sharp events are mimicked in the lab using step-like (e.g., Simons, 1978; Stüttgen et al., 2006) or pulse-like (e.g., Garabedian et al., 2003; Gerdjikov et al., 2010; Stüttgen and Schwarz, 2010) whisker deflections. Both, active whisker touches as well as their experimental substitutions, are well known to lead to a single spike or short burst of spikes typically followed by a long lasting inhibition (Simons, 1985; Hentschke et al., 2006; Stüttgen and Schwarz, 2008; Jadhav et al., 2009). The horizontal spread of such responses is exceedingly wide, covering at least the whole barrel field and even reaching far distant cortical areas in the same hemisphere (Ferezou et al., 2007). Intracortical microstimulation shows characteristics quite similar to those seen with the more natural whisker-mediated touch. Intracortical microstimulation-evoked responses consist in single spikes or short bursts and spread in horizontal direction surely reaching adjacent columns and columns further afield (Butovas and Schwarz, 2003). Notably, direct comparison of voltage sensitive signals evoked by ICMS and mechanical whisker deflection resulted in very similar spatio-temporal activation patterns across the cortex of the entire hemisphere in mice (Ferezou et al., 2007). Further, like touch-evoked activity, local ICMS excitatory responses are followed by a long inhibitory response lasting for about 100 ms (Butovas and Schwarz, 2003; Butovas et al., 2006). Perhaps the most obvious difference between whisker deflection and ICMS-evoked responses is that the former show strong response adaptation in the barrel cortex (Chung and Ferster, 1998; Garabedian et al., 2003; Khatri et al., 2004; Stüttgen and Schwarz, 2010), while repetitive ICMS reliably evokes excitatory responses in barrel cortex due to direct activation of axons (Butovas and Schwarz, 2003). If anything, this should ease the use of frequency for the purpose of discrimination. In fact, repetitive ICMS improves detection but perceptual gains are maximal with short bursts of stimulation pulses (above 40 Hz). This suggests that adaptation and/or evoked inhibition play a role in limiting the responses of downstream cortical areas (Butovas and Schwarz, 2007). In the present study temporal frequencies of repetitive ICMS are typically around 60 Hz (three pulses per protraction lasting ~50 ms) and therefore should be well within this optimal perceptual window. Taken together, based on these known facts there is no reason why the rats in the present experiments should not be able to exploit the spatial frequency of virtual stimuli for discrimination.

Our finding that rats have great difficulties to use the frequency cues is supported by evidence that these animals typically do not integrate passively presented repetitive whisker deflections—neither for detection (Stüttgen and Schwarz, 2010) nor for discrimination purposes (Waiblinger et al., 2013). In the first cited study, detection of repetitive pulses was worse than expected from response probabilities to single pulses, exactly what has been found with ICMS pulses (Butovas and Schwarz, 2007). Further, a probabilistic model predicted the detection performance of rats very well, but only if integration constants of 5–8 ms were used (Stüttgen and Schwarz, 2010). In the Waiblinger et al. (2013) study, rats showed a poor performance if repetitive pulsatile discriminanda exclusively contained frequency and intensity cue but not amplitude cues. Whenever amplitude cues were present rats discriminated superiorly. Our present results with active touch match these earlier results. Given the difficulties of the rats to discriminate spatial frequency, we did not try to train them on a psychometric task aimed at measuring frequency thresholds of discrimination. Our rats first failed and then, with a specific strategy to guide them towards discriminating spatial frequency, they achieved a mediocre discrimination performance when other stimuli containing ICMS amplitude cues were presented in the same session (Test 2). Only after learning this they could be confronted with a task that required discrimination based on frequency cues for all presented stimuli (Test 3). They initially performed well but could sustain their performance only for a short period a few hundred trials. The short initial performance was only visible when analyzing their performance using a moving window (in session-wise assessment of performance it was largely averaged out). In summary, together with the earlier results from passive touch, we conclude that frequency is not a major factor on which rats base vibrotactile discrimination. The consistent overall degradation of performance on frequency cues consistently seen in all animals clearly shows the limitations of the rats to sustain sufficient discrimination based on frequency cues. Our failure to detect any systematic attempt of the rats to adjust whisking parameters in any of the tests, strongly supports this conclusion.

How does this conclusion compare with what is known about coding in tactile systems of other species, notably primates? In fact, macaques are able to passively discriminate long strips of sinusoids and pulsatile stimuli (LaMotte and Mountcastle, 1975; Mountcastle et al., 1990; Hernández et al., 1997; Salinas et al., 2000). It is important to note that in these studies stimuli of different frequency were amplitude-adjusted for differences in “subjective intensity”. These adjustment served to bolster the statement that in those tasks the subjects used frequency, not intensity, as the basis for their perceptional decision. It needs to be borne in mind that these classic experiments were not designed to find out about the coding of instantaneous kinematic parameters. Obviously, the stimuli matched for subjective intensity displayed marked differences in instantaneous kinematic cues, which the animals in principle might have employed to take their decision. In fact, at present, there are no published experiments in primates conclusively addressing the possible role of kinematic event coding for tactile perception. Caution in adopting a final view in this matter is further suggested by research in the monkey visual system where results from reaction time tasks (Roitman and Shadlen, 2002; Kiani et al., 2008; Cohen and Newsome, 2009) have cast strong doubt on the classic idea that temporal integration of visual signals for perceptual decision making is in principle unlimited (Britten et al., 1992).

In rats, an important role for coding of kinematic events has been revealed by systematic variation of amplitude and frequency of pulsatile discriminanda (Waiblinger et al., 2013). Further, it is a long-known fact that increasing amplitude/velocity of whisker deflections results in increasing activity in barrel cortex neurons (Simons, 1978; Stüttgen and Schwarz, 2010). Considering that this increment is paralleled by stronger responses with increased amplitudes of ICMS, we argue that ICMS current amplitude in the present virtual active touch experiment might have assumed the role played by whisker deflection amplitude/velocity in the passive touch study (Waiblinger et al., 2013). Accepting this conjecture points to amplitudes of whisker deflection as the dominant cue for vibrotactile discrimination also in the active case. Future studies are needed to elucidate whether and how rats store detailed kinematic events in memory and which role they play in the adaptation of whisking strategies to optimize perception.

## Author contributions

Petya Georgieva conceived experiments, conducted experiments, analyzed data, wrote the paper. Dominik Brugger conceived experiments, established the experimental setup. Cornelius Schwarz conceived the experiments, analyzed data, wrote the paper.

## Conflict of interest statement

The authors declare that the research was conducted in the absence of any commercial or financial relationships that could be construed as a potential conflict of interest.
